# Dependence of lattice strain relaxation, absorbance, and sheet resistance on thickness in textured ZnO@B transparent conductive oxide for thin-film solar cell applications

**DOI:** 10.3762/bjnano.7.9

**Published:** 2016-01-20

**Authors:** Kuang-Yang Kou, Yu-En Huang, Chien-Hsun Chen, Shih-Wei Feng

**Affiliations:** 1 Department of Traffic Science, Central Police University, Taoyuan, Taiwan; 2 Department of Applied Physics, National University of Kaohsiung, No.700 Kaohsiung University Road, Nan-Tzu Dist., 811. Kaohsiung, Taiwan,; 3 Green Energy and Environment Research Labs, Industrial Technology Research Institute, Hsinchu, Taiwan

**Keywords:** absorbance, low-pressure chemical vapor deposition, strain relaxation, transparent conductive oxide, textured ZnO

## Abstract

The interplay of surface texture, strain relaxation, absorbance, grain size, and sheet resistance in textured, boron-doped ZnO (ZnO@B), transparent conductive oxide (TCO) materials of different thicknesses used for thin film, solar cell applications is investigated. The residual strain induced by the lattice mismatch and the difference in the thermal expansion coefficient for thicker ZnO@B is relaxed, leading to an increased surface texture, stronger absorbance, larger grain size, and lower sheet resistance. These experimental results reveal the optical and material characteristics of the TCO layer, which could be useful for enhancing the performance of solar cells through an optimized TCO layer.

## Introduction

Thin-film solar cells require a transparent conductive oxide (TCO) to allow light to reach the absorber layers and create the electrical current. Due to its superior characteristics, including a wide band gap, high dielectric constant, high exciton binding energy (60 meV), high thermal stability, high transparency, and high conduction, wurtzite ZnO is a very promising TCO material used for the front contact, barrier layer, and intermediate reflector in solar cells [1–9]. Low-pressure chemical vapor deposition (LPCVD) can be implemented to deposit such a transparent, textured, and highly conductive TCO [9]. For thin-film solar cell applications, the LPCVD-grown ZnO can possess an as-grown textured structure to enhance light scattering and to increase the optical path through the solar cell without any post-treatment. Because the structural, optical, and electrical characteristics of LPCVD-grown ZnO are sensitive to the growth temperature, pressure, and flow rate, TCO can be tuned according to the application.

ZnO films grown on a sapphire substrate undergo residual strain induced by the lattice mismatch and the difference in thermal expansion coefficient [10]. Because strain can affect the electronic and optical properties of materials, the strain distribution in the films is an important subject to be investigated. The strain in ZnO films is accumulated during film growth and can be monitored by in situ optical reflectance measurement as a function of thickness, *t*: compressive strain for *t* < 5.5 nm, released compressive strain due to generation of misfit dislocations for 5.5 nm < *t* < 200 nm, tensile strain due to thermal stress for 200 nm < *t* < 500 nm, and residual tensile strain relaxed by microcrack formation for *t* > 500 nm [10].

The variation in the physical properties of nanostructures drastically influences the optoelectronic properties of ZnO [11–13]. X-ray-excited optical luminescence of ZnO nanoneedles shows a sharp band gap emission and a broad red emission related to surface defects, while that of ZnO microcrystallites has a strong green emission due to defect states in the core [11]. A blue-shifted absorption edge and photoluminescence caused by quantum confinement as well as a higher photovoltaic and sensor performance due to a larger surface area have been demonstrated in ZnO nanocrystals [14–16]. In addition, the microstructure, optical properties, and strain of thickness-dependent ZnO thin film grown by atomic layer deposition have been reported [17]. The thicker ZnO thin films show a larger crystalline grain, a smaller lattice strain, a higher Zn/O ratio, and better crystal quality. Furthermore, doping impurities, such as B, Al, and Ga, can improve the electrical transport properties of ZnO [18]. There are two benefits to using a B-doped ZnO (ZnO@B) film as the TCO layer. First, B has the smallest ionic radius among the three dopants (B^3+^: 0.23Å, Al^3+^: 0.54 Å, Ga^3+^: 0.62 Å,), which results in better transparency. Second, a textured surface can be easily achieved for a ZnO@B film grown by LPCVD, which can enhance light scattering and increase the optical path through the solar cell without any post-treatment. However, many important issues regarding optical and material characteristics in textured, ZnO@B TCO grown by LPCVD for thin-film solar cell applications is yet to be explored.

This study reports the lattice strain relaxation, absorbance, and sheet resistance of textured ZnO TCO@B for solar cell applications. A thicker ZnO@B film enhances the strain relaxation, resulting in an increased surface texture, stronger absorbance, larger grain size, and lower sheet resistance. The optimization of the TCO layer could be useful for enhancing the performance of solar cells.

## Results and Discussion

### Structural characterization

**Figure 1:**
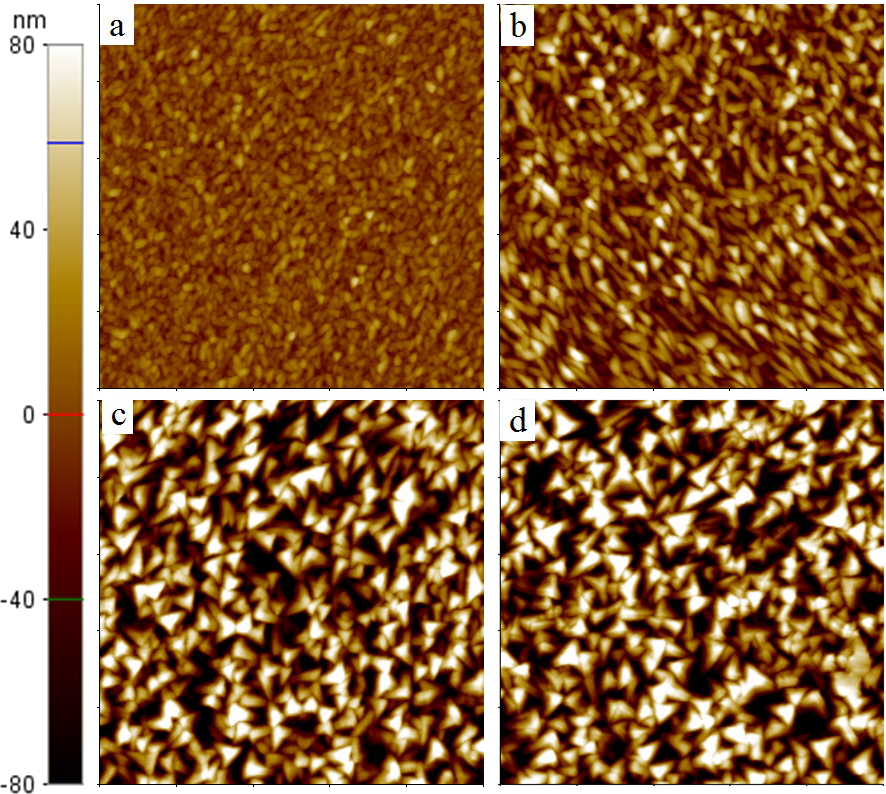
AFM images (5 × 5 μm) of (a) *c*-20 (16.603 nm), (b) *c*-40 (26.756 nm), (c) *c*-60 (51.531 nm), and (d) *c*-70 (56.233 nm) ZnO@B samples. The surface roughness of each sample is given in the parentheses.

Four ZnO@B samples with 20-, 40-, 60-, and 70-minute growth times were prepared (here within named, *c-*20, *c-*40, *c*-60, and *c-*70, respectively). Figure 1 shows the atomic force microscopy (AFM) images (5 × 5 μm) of the four ZnO@B samples. The surface roughness is 16.603, 26.756, 51.531 and 56.233 nm for *c*-20, *c*-40, *c*-60, and *c*-70, respectively. Each sample is composed of small grains. The grain size of the triangular features is estimated to be 676, 1520, 2706, and 4220 nm^2^ for *c*-20, *c*-40, *c*-60, and *c*-70, respectively. As the growth time (thickness) increases, the surface becomes more textured and the grain size larger. The film is composed of vertically stacked monocrystalline grains that appear as pyramids at the surface. The apparent texture structure in the sample *c*-70 can effectively scatter light to enhance the light coupling.

**Figure 2:**
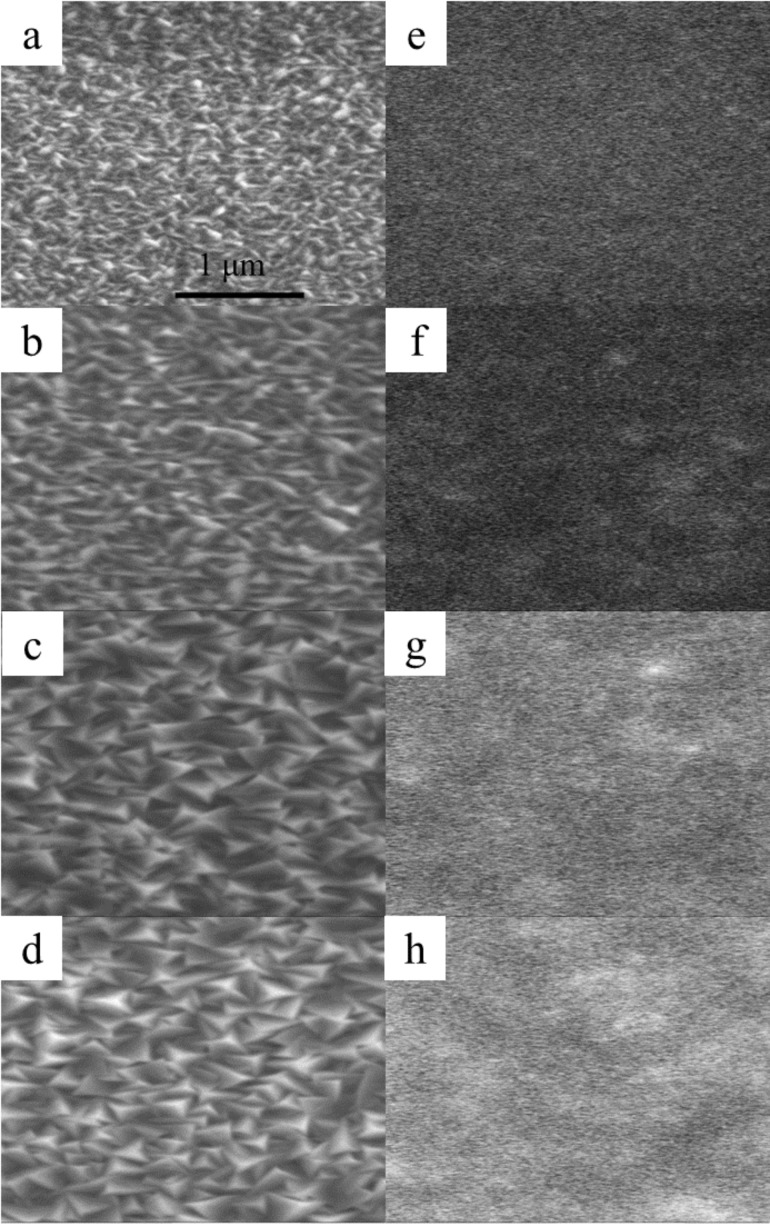
SEM images of the (a) *c*-20, (b) *c*-40, (c) *c*-60, and (d) *c*-70 ZnO@B samples and the panchromatic CL images (e), (f), (g), and (h), respectively, taken over the same regions with 11 kV excitation electron voltage at RT.

Figure 2a–d shows scanning electron microscope (SEM) images of the *c*-20, *c*-40, *c*-60, and *c*-70 ZnO@B samples, respectively. Figure 2e–h shows the panchromatic cathodoluminescence (CL) images of the corresponding SEM regions using an 11 kV excitation electron voltage. In the SEM images, the *c-*20 sample is composed of small grains with no preferential orientation. Increasing thickness leads to an apparent textured structure and larger grain size. The CL image of the *c*-70 sample shows a high contrast and bright image, while that of the *c*-20 shows a low contrast and dark one. This shows that the apparent texture structure enhances light scattering.

**Figure 3:**
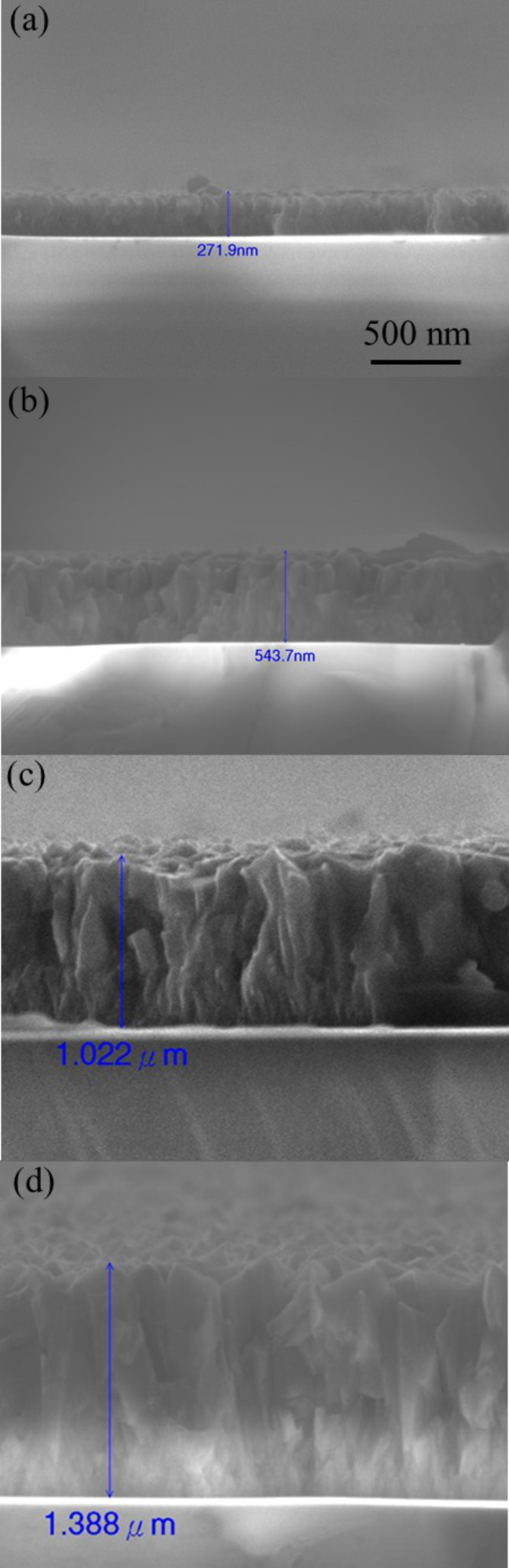
Cross-sectional SEM images of the (a) *c*-20, (b) *c*-40, (c) *c*-60, and (d) *c*-70 ZnO@B samples. The thickness of each sample is shown in the image.

Figure 3a–d shows show the cross-sectional SEM images for the *c*-20, *c*-40, *c*-60, and *c*-70 ZnO@B samples, respectively. From the cross-sectional SEM images of each sample, the thicknesses of the *c*-20, *c*-40, *c*-60, and *c*-70 samples are estimated to be 271.9, 543.7, 1,022, and 1388 nm, respectively.

### Optical properties

**Figure 4:**
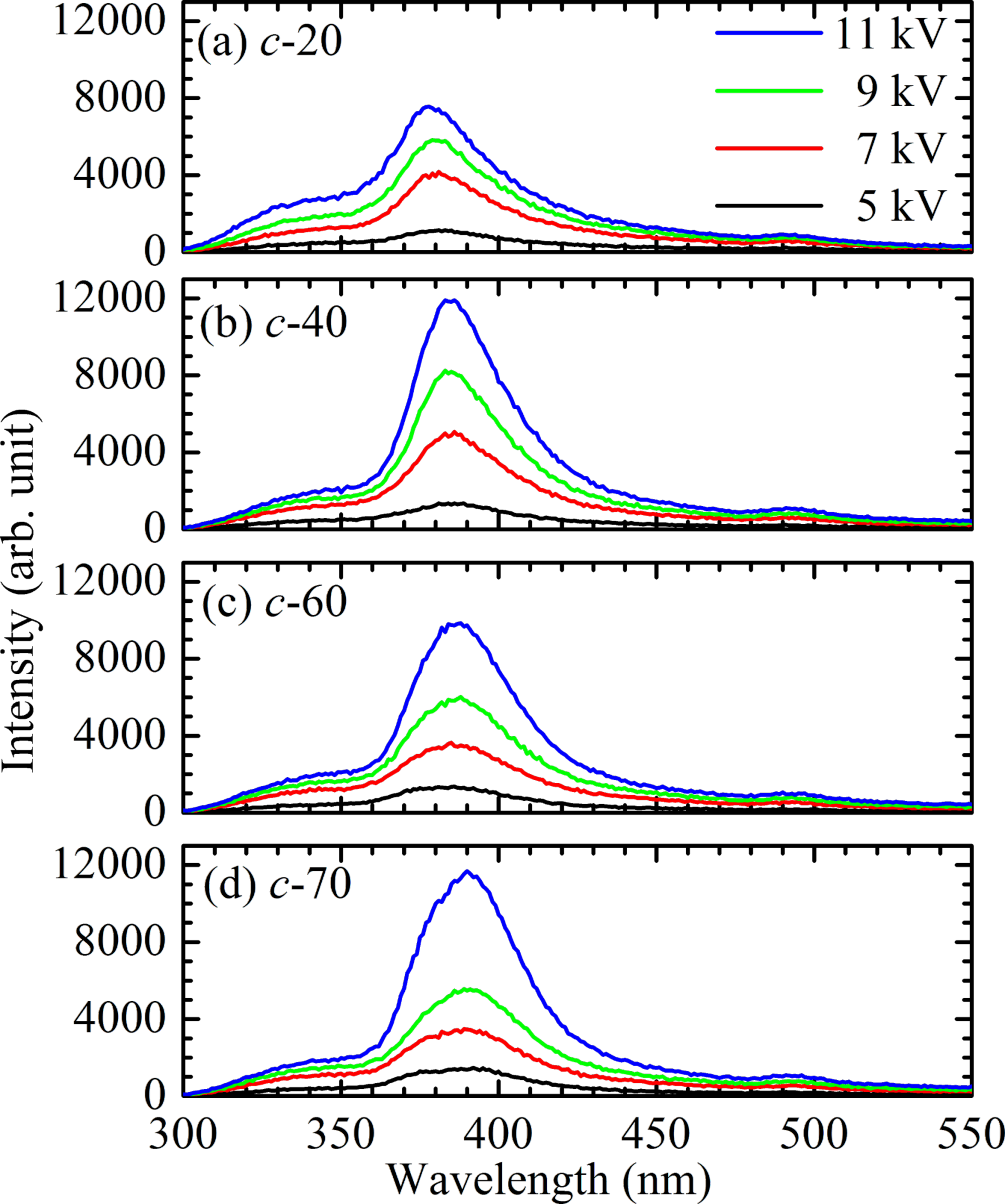
CL spectra of the (a) *c*-20, (b) *c*-40, (c) *c*-60, and (d) *c*-70 ZnO@B samples for excitations of 5, 7, 9, and 11 kV at RT.

Figure 4 shows the CL spectra of the four samples at room temperature (RT). As the thickness increases, the CL emission peak is slightly red-shifted. An emission peak around 378 nm (3.28 eV) is related to a band-to-band transition, while that in the 382–386 nm (3.21–3.24 eV) spectral range is due to transitions between band-tail states of ZnO [17]. Hence, as the thickness increases, the dominant red-shifted CL emission moves from the band-to-band transition to the transition between the band-tail states. Due to a larger grain size in the thicker *c*-70 sample, the red-shifted CL emission is consistent with the weaker quantum size effect.

**Figure 5:**
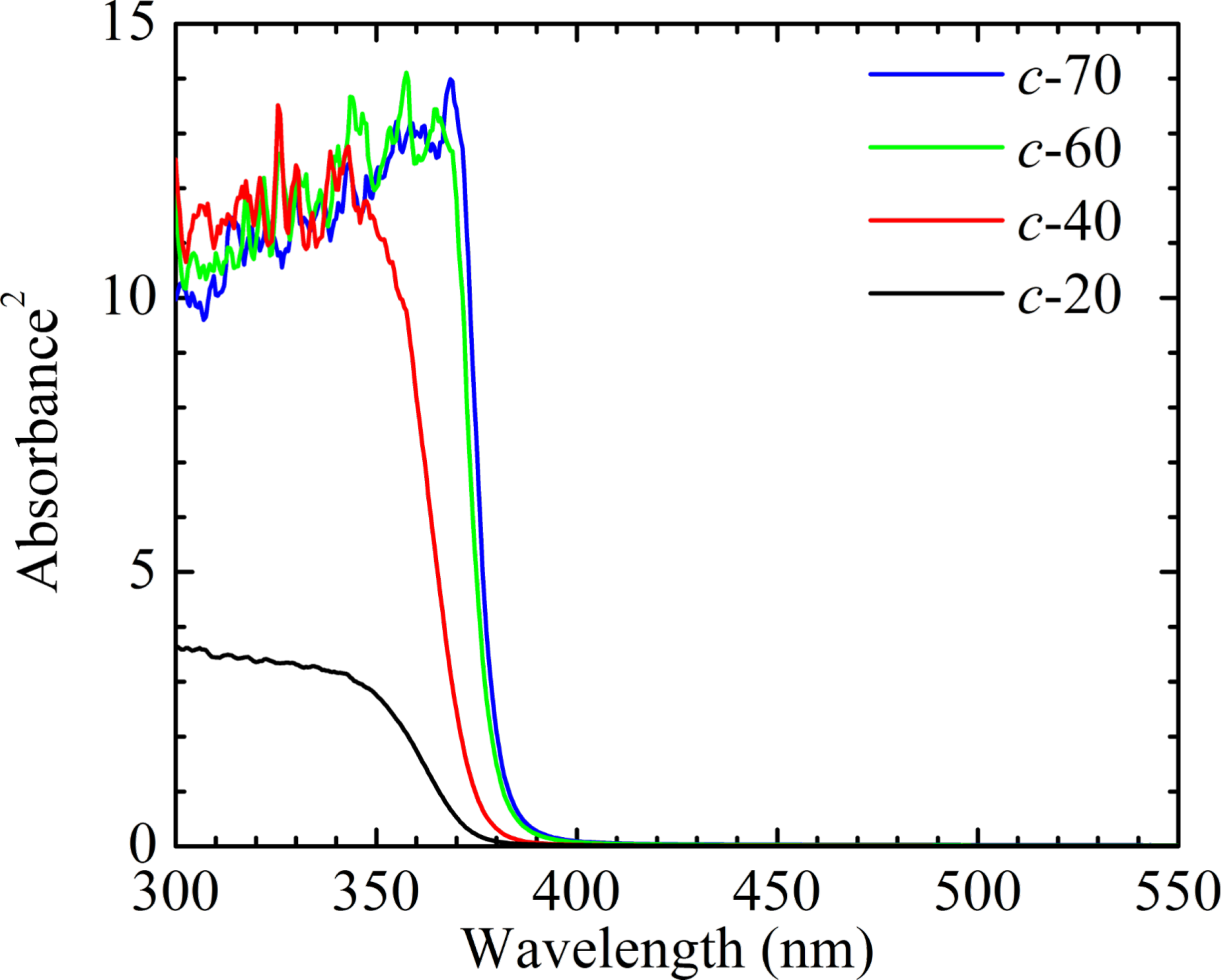
Absorbance (squared) of the four ZnO@B samples.

Figure 5 shows the absorbance squared, α^2^(*E*), of the four samples. Compared to the *c*-20 sample, the other three samples show enhanced absorbance in the 300–350 nm spectral range. As the thickness increases, the sharper, red-shifted absorption edge in the 360–380 nm spectral range is consistent with the more uniform distribution of grain size and weaker quantum size effect. The band gap energy, *E*_g_, of a semiconductor can be estimated by extrapolating the linear portion of the absorbance square to zero. As shown in Table 1, as the thickness increases, the red-shifted *E*_g_ is consistent with the weaker quantum size effect. The lower *E*_g_ of ZnO@B samples compared to that of Al-doped ZnO (AZO or ZnO@Al) samples is beneficial for photon absorption and contributes to the quantum efficiency of solar cells [19].

**Table 1:** Thickness (nm), grain size (nm^2^), roughness (nm), *E*_g_ (eV), Δω(*A*_1_(TO)) (cm^−1^), ε (%), and sheet resistance (*R*_sheet_) (ohm/□) for the four ZnO@B samples.

Sample	Thickness (nm)	Grain size (nm^2^)	Roughness (nm)	*E*_g_ (eV)	Δω(*A*_1_(TO)) (cm^−1^)	ε (%)	*R*_sheet_ (ohm/□)

*c*-20	272	696	16.603	3.32	−0.082	0.0069	846
*c*-40	544	1,220	26.756	3.31	−0.809	0.0682	191
*c*-60	1,022	2,706	51.531	3.28	−1.534	0.1292	96
*c*-70	1,388	4,220	56.233	3.26	−0.082	0.0069	76

### Strain relaxation

**Figure 6:**
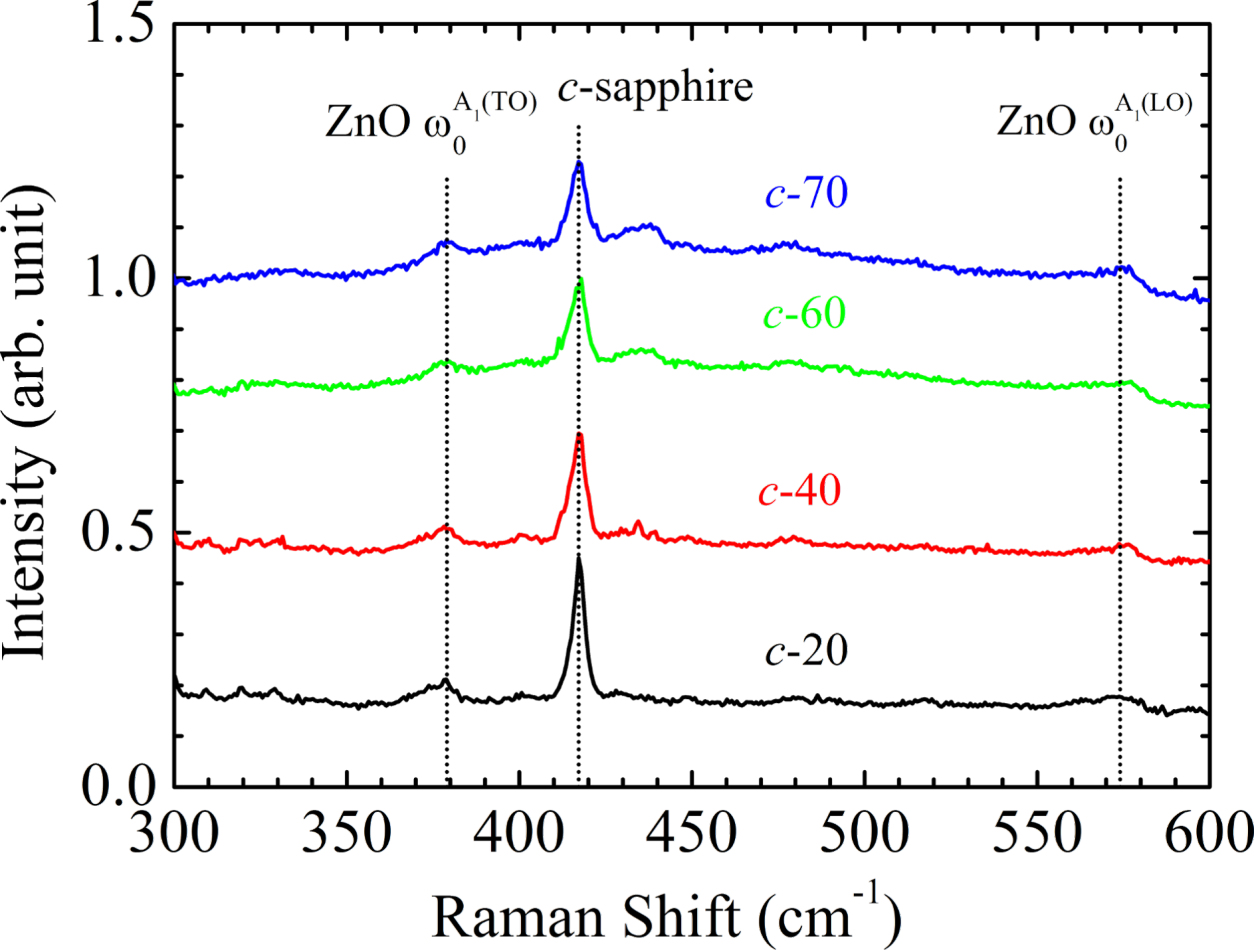
Raman scattering spectra of the four ZnO@B samples. The spectra display *A*_1_(TO) and *A*_1_(LO) modes for ZnO. The dotted lines at 379 cm^−1^ and 574 cm^−1^ show the strain-free *A*_1_(TO) and *A*_1_(LO) modes, respectively, for ZnO [20].

To study the effect of thickness on strain relaxation, Raman measurements were taken to estimate the in-plane strain of the four samples. Figure 6 shows the Raman scattering spectra for the four samples. The spectra display *A*_1_(TO) and *A*_1_(LO) modes for ZnO. The dotted lines at 379 cm^−1^ and 574 cm^−1^ show the strain-free *A*_1_(TO) and *A*_1_(LO) modes, respectively, for ZnO [20]. The in-plane strain in the *x*-direction, ε*_xx_*, and in the *y*-direction, ε*_yy_*, of ZnO films can be determined by the frequency shift, Δω = ω−ω_0_ [21], as:

[1]
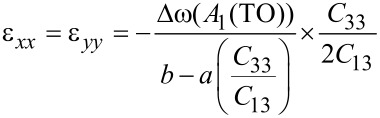


where *a* = −774 cm^−1^ and *b* = −375 cm^−1^ are the deformation potential constants of the *A*_1_(TO) mode [22]. The elastic stiffness constants, *C*_33_ and *C*_13_, are 216 and 104 GPa, respectively [1]. The six-fold symmetry of the hexagonal polar *c*-ZnO dictates an isotropic in-plane strain in the basal plane, i.e., ε*_xx_* = ε*_yy_* = ε. The frequency shift,s Δω, for the *A*_1_(TO) mode are shown in Table 1. The in-plane strain ε can be deduced from Equation 1.

**Figure 7:**
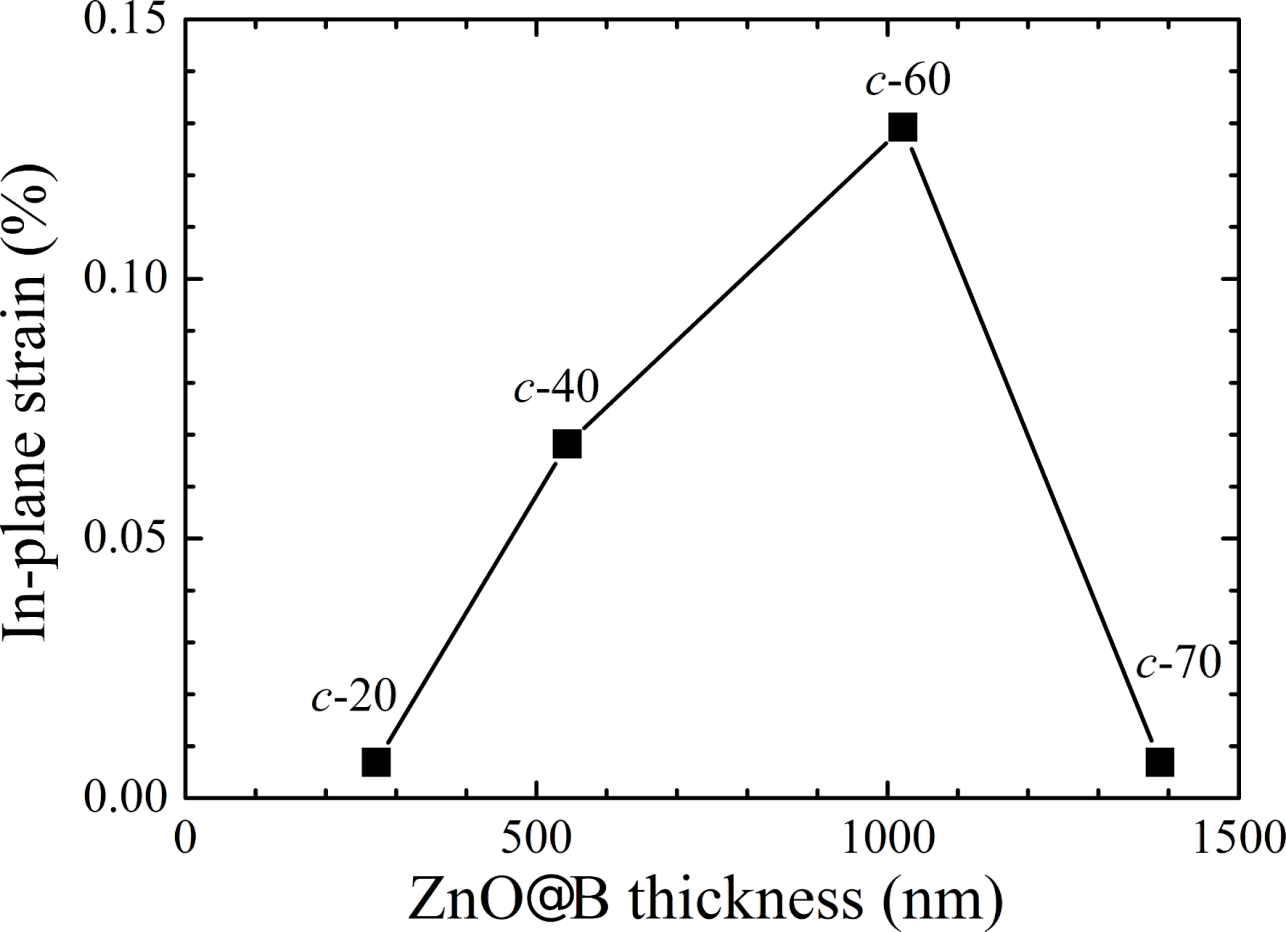
In-plane strain for the four ZnO@B samples as a function of ZnO thickness.

The in-plane tensile strain, ε, of the *c*-20, *c*-40, *c*-60, and *c*-70 samples is 0.0069, 0.0682, 0.1292, and 0.0069%, respectively. Figure 7 shows the evolution of in-plane strain for the four samples as a function of thickness. As the thickness increases, the in-plane tensile strain becomes larger and then relaxed. A similar trend, attributed to strain relaxation by microcrack formation, is also observed in the ZnO thin film grown by plasma-assisted molecular-beam epitaxy [10]. Therefore, for textured, ZnO@B TCO, the residual strain induced by the lattice mismatch and the difference of thermal expansion coefficient is relaxed, leading to an apparent textured surface and larger grain size. The relaxed strain in the *c*-70 sample improves the sample quality, showing a lower sheet resistance. The lower sheet resistance and increased surface texture for the *c*-70 sample may represent the best TCO candidate. However, the stronger absorbance in the UV spectral range in the thicker TCO layer could decrease the quantum efficiency of the thin-film solar cell. To utilize the ZnO@B TCO on solar cells, the TCO layer must be grown on a solar cell and its optimal thickness thus could be chosen to match the overall material and structure of the cell.

## Conclusion

In summary, a thicker, ZnO@B TCO layer enhances the strain relaxation and hence shows an increased surface texture, stronger absorbance, less transparence, larger grain size, and lower sheet resistance. For solar cell applications, the TCO layer should be highly transparent and less resistive. Although the smaller sheet resistance and increased surface texture in thicker ZnO@B samples are beneficial to device performance, photons in the UV spectral range are strongly absorbed in such a TCO layer and could limit the quantum efficiency of the thin-film solar cell. For the application of ZnO@B TCO in solar cells, the TCO layer must be grown on a solar cell and its optimal thickness therefore could be chosen to match the overall material and structure of the cell for enhancing the performance of solar cells. Nevertheless, this is an interesting and important subject which will be left for future investigation.

## Experimental

### Synthesis of ZnO@B thin films by LPCVD

The polar *c*-plane ZnO samples were grown on a sapphire substrate at 170 °C at a pressure of 0.6 Torr in a LPCVD reactor. DEZ and H_2_O were used as the precursors for Zn and O, respectively. ZnO was doped with B to improve the electrical transport properties of ZnO thin ﬁlms [9,18]. B_2_H_6_ was used as a doping gas. Four ZnO@B samples with 20-, 40-, 60-, and 70-minute growth times, corresponding to 272, 544, 1022, and 1388 nm in thickness, were prepared (samples *c-*20, *c-*40, *c*-60, and *c-*70, respectively). For the four samples, the B_2_H_6_, DEZ, and H_2_O flow rates were 1, 500, and 550 sccm, respectively.

### Characterization

The surface morphology was revealed by atomic force microscopy (Park Systems, XE-70) performed in noncontact mode using a silicon tip with a curvature of less than 10 nm. The scanning electron microscope and cathodoluminescence results were acquired by the use of a Gatan monoCL3 spectrometer in a JEOL JSM 7000F SEM system. The absorption spectra were acquired with a U-3900 spectrophotometer (model 2J2-0015) at room temperature. The Raman spectra were recorded in the backscattering configuration using a Jobin Yvon-Horiba micro-Raman system (model T64000) with a 532 nm laser.
